# Identifying levels of alcohol use disorder severity in electronic health records

**DOI:** 10.1186/s13011-025-00670-w

**Published:** 2025-09-08

**Authors:** Jakob Manthey, Carolin Kilian, Ludwig Kraus, Ingo Schäfer, Anna Schranz, Bernd Schulte

**Affiliations:** 1 Centre for Interdisciplinary Addiction Research (ZIS), Department of Psychiatry and Psychotherapy, University Medical Center Hamburg-Eppendorf (UKE), Martinistraße 52, 20246 Hamburg, Germany; 2 Department of Psychiatry, Medical Faculty, University of Leipzig, Semmelweisstraße 10, 04103 Leipzig, Germany; 3 National Institute of Public Health, University of Southern Denmark, Copenhagen, Denmark; 4 Danish Institute for Advanced Studies (DIAS), University of Southern Denmark, Odense, Denmark; 5 Department of Public Health Sciences, Centre for Social Research on Alcohol and Drugs, Stockholm University, Stockholm, Sweden; 6 Institute of Psychology, ELTE Eötvös Loránd University, Budapest, Hungary

**Keywords:** Alcohol use disorder, Electronic health records, Comorbidity, ICD-10, Epidemiology

## Abstract

**Background:**

Alcohol use disorder (AUD) is conceptualized as a dimensional phenomenon in the DSM-5, but electronic health records (EHRs) rely on binary AUD definitions according to the ICD-10. The present study classifies AUD severity levels using EHR data and tests whether increasing AUD severity levels are linked with increased comorbidity.

**Methods:**

Billing data from two German statutory health insurance companies in Hamburg included *n* = 21,954 adults diagnosed with alcohol-specific conditions between 2017 and 2021. Based on ICD-10 alcohol-specific diagnoses, patients were classified into five AUD severity levels: 1 (F10.0, T51.0 or T51.9); 2 (F10.1); 3 (F10.2); 4 (F10.3/4); 5 (K70 + or one of the following diagnoses: K70.0-4, K70.9, K85.2, K85.20, K86.0, 10.5-9, E24.4, G31.2, G62.1, G72.1, I42.6, K29.2). Generalized estimating equation regression models for count data (Poisson distribution) were used to assess associations with the Elixhauser Comorbidity Score (ECS).

**Results:**

Across the study period, the annual prevalence of any AUD diagnosis varied between 2.7% and 2.9%. A dose-response relationship was observed between AUD severity and ECS, indicating that individuals with higher AUD severity experience more comorbid conditions, particularly cardiovascular and liver diseases.

**Conclusions:**

The proposal to define AUD severity levels based on ICD-10 diagnoses allows for a more nuanced analysis of AUD in EHR data.

**Supplementary Information:**

The online version contains supplementary material available at 10.1186/s13011-025-00670-w.

## Background

Alcohol use is prevalent globally [1] and causally linked to the deterioration of mental and physical health [2], resulting in sizeable health losses [3]. Epidemiological approaches to investigating the prevalence and distribution of use rely heavily on self-reported measurements, i.e., general population surveys [1]. However, surveys tend to overrepresent healthier populations, limiting their use to describe those drinking heavily and experiencing alcohol-attributable harms [4]. Overcoming biases inherent in survey data and triangulation with sales data [5] allows a comprehensive assessment of alcohol-attributable harms in a given jurisdiction [6].

In addition to these ‘traditional’ methods, electronic health records (EHR) are increasingly used to capture the population affected by alcohol use disorders (AUD). In various jurisdictions, EHR has been shown to provide a feasible approach to measuring the comorbidity of people affected by AUD [7, 8] as well as identifying predictors of re-admission [9] and mortality risks [10].

In many high-income countries, EHR is available for a large share of the population, e.g., when derived from national registries or large health insurance companies. This data may exhibit minimal selection biases and provide a high-resolution of diagnostic information. Despite these advantages, the use of EHR in alcohol epidemiology is underexplored, possibly due to the lack of data on alcohol exposure and insufficient socioeconomic details. To increase the use of EHR, limitations inherent to the data need to be acknowledged and possibly overcome.

In our view, a key use case of EHR in the context of alcohol epidemiology is a detailed characterization of people with AUD and their health burden. To achieve this, a clear and reproducible definition of AUD is required, but currently not established in EHR data. In the DSM-5, AUD is considered a dimensional rather than a binary phenomenon, which also reflects the dose-response relationship between alcohol intake and many disease outcomes, including AUD diagnoses like alcohol dependence [2, 11]. Moreover, conceptualizing AUD as a continuum that is mainly determined by the level of chronic alcohol intake over time was expected to reduce stigmatization towards those affected [12]. Other attempts to assess the dimensionality of AUD have relied on symptom or criterion counts (see e.g. [13, 14]), but we are not aware of any dimensional approach using EHR data. Moreover, a dimensional AUD concept is not incorporated in the ICD-10 – the prevailing diagnostic system in healthcare in most countries. This currently limits our understanding of the differential needs of people with AUD. However, dozens of ICD-10 diagnoses describe the harm caused by different patterns and levels of alcohol intake. With a hierarchical order of ICD-10 diagnoses that correlate with both increasing levels of alcohol intake, we propose the following 5-level AUD severity dimension.

Consistent with a distinct three-stage model of addiction [15], a single heavy alcohol use episode constitutes AUD severity level 1. Depending on the prevailing coding practices, these single episodes are diagnosed with F10.0 (acute intoxication) or T51 (toxic effect of alcohol). AUD severity level 2 reflects harm from continued alcohol use and is diagnosed with F10.1 (harmful use). Those continuing to use alcohol despite experiencing harm can experience loss of control and increased tolerance to alcohol. This would be AUD severity level 3 and is diagnosed with F10.2 (dependence). AUD severity level 4 can occur among those with alcohol dependence discontinuing their alcohol use, who experience a state of withdrawal [16]. It is diagnosed with F10.3 (withdrawal) or F10.4 (withdrawal with delirium). Lastly, the chronic heavy use of alcohol over a longer time, often more than one or two decades, can result in alcohol-induced psychotic disorder and amnesia (F10.5 to F10.9), alcoholic liver cirrhosis (K70), alcoholic cardiomyopathy (I42.6), alcoholic pancreatitis (K85.2 and K86.0) or alcoholic gastritis (K29.2), which constitutes AUD severity level 5.

This classification aims to provide a system similar to the DSM-5 AUD severity groupings, approximating disease progress at a population level rather than providing a unique, person-specific assessment of different, possibly co-occurring, clinical states of AUD. The proposed classification considers alcohol dependence as the more severe manifestation of alcohol problems than harmful use, which is consistent with the definitions in the diagnostic system [17]. Moreover, alcoholic cardiomyopathy and liver cirrhosis are the result of long-term heavy alcohol use [18, 19] and thus are considered indications of the most severe form of AUD. In contrast to these markers of chronic disease progression, the proposed classification also draws on diagnoses referring to clinical states, such as single heavy alcohol use episodes or withdrawal. For people with a single heavy alcohol use episode and no other alcohol-specific diagnosis, the first of three distinct stages of addiction [15] may be assumed. For people with alcohol withdrawal syndrome, we assume greater AUD severity. Early experimental research has demonstrated that symptoms are linked to alcohol intake in a dose-response relationship [20], and withdrawal symptoms were closely linked to AUD severity in general population samples [13, 21]. To group withdrawal syndrome into the classification system, we examined incidence frequencies: alcohol withdrawal syndrome is a common phenomenon among people with AUD (50% according to [16]), while fewer people with AUD experience alcoholic liver disease (meta-analysis: 12.9%; [22]). Thus, we consider that alcohol withdrawal syndrome constitutes a somewhat lower AUD severity level than alcoholic liver disease.

In this contribution, we (1) describe the proposed AUD severity levels based on the ICD-10 diagnostic system. Using this definition, we (2) aimed to estimate the administrative prevalence of AUD according to varying severity levels and (3) tested whether increasing AUD severity levels are linked to increased comorbidity, as demonstrated previously [23].

## Methods

### Data sources

Billing data was provided by two German statutory health insurance companies (SHI; AOK Rheinland/Hamburg - Die Gesundheitskasse; DAK – Gesundheit). In Germany, billing data contains information on the management and treatment of patients and is sent to the SHI by outpatient health practitioners and hospitals. Thus, billing data is an integral part of EHR and is commonly used in healthcare service research in Germany.

The Federal Office for Social Security approved the conduct of this study, including data linkage. This approval exempted the study from seeking formal ethics approval, as we had no access to personal identifying information at any time, but only handled and analysed pseudonymized electronic health records. To pseudonymize the data, the data owners deleted the patient identifier and generated a unique identifier that made it possible to link the data with other sources (see study protocol [24]).

### Study population and diagnoses considered

The study population included all persons aged 18 years or older (upper recorded age limit: 99 years) insured at any time between 2017 and 2021 with an alcohol-specific diagnosis (see Table1) in outpatient (e.g., general practitioner, psychiatrist) or inpatient (i.e., hospital, including emergency department) settings. At least one day of health insurance in a given year was required to be included in year-specific analyses.

In Germany, different types of diagnoses are given in both outpatient and inpatient settings. We included ‘confirmed’ diagnoses in outpatient (as opposed to ’exclusionary’, ’suspecting’, and ’symptomless’ diagnoses; less than 10% of all alcohol-specific outpatient diagnoses) and ‘admission’, ‘primary’, and ‘secondary’ diagnoses in inpatient settings (as opposed to surgery diagnoses or follow-up diagnoses; less than 4% of all alcohol-specific inpatient diagnoses). Diagnoses in outpatient settings are registered quarterly, and we used the median day of each quarter to determine the date of each diagnosis. For diagnoses in inpatient settings, the first and last dates are registered, and we used the first day to determine the date of each inpatient diagnosis.

**Table 1:** Definition of AUD severity levels

Domain	Level	Definition
AUD severity: Type of alcohol-specific diagnosis	0 (any)	Any alcohol-specific diagnosis
	1 (F10.0)	Alcohol intoxication: At least one of the following diagnoses: F10.0, T51.0, T51.9
	2 (F10.1)	Harmful alcohol use: At least one F10.1 diagnosis
	3 (F10.2)	Alcohol dependence: At least one F10.2 diagnosis
	4 (F10.3/4)	Alcohol withdrawal without or with delirium
	5 (K70+)	Alcoholic liver cirrhosis or other diseases resulting from chronic heavy alcohol use over several years; at least one of the following diagnoses: K70.0, K70.1, K70.2, K70.3, K70.4, K70.9, K85.2, K85.20, K86.0 F10.5, F10.6, F10.7, F10.8, F10.9, E24.4, G31.2, G62.1, G72.1, I42.6, K29.2

We considered all ICD-10 diagnoses indicating occasional or chronic heavy use of alcohol (F10.0, F10.1, F10.2, F10.3, F10.4, F10.5, F10.6, F10.7, F10.8, F10.9, E24.4, G31.2, G62.1, G72.1, I42.6, K29.2, K70.0, K70.1, K70.2, K70.3, K70.4, K70.9, K85.2, K85.20, K86.0, T51.0, T51.9). We did not include alcohol-specific diagnoses indicating fetal damage due to maternal alcohol use (O35.4; *n* = 2 diagnoses) or the mere presence of alcohol in the blood (R78.0; *n* = 80 diagnoses).

### AUD severity

As summarized in Table 1, AUD severity was defined based on the type of alcohol-specific diagnosis. We classified four partially overlapping severity groupings, i.e., a person with an F10.2 *and* F10.3 diagnosis was included in groups 3 and 4. One additional overarching group (“0”) included all persons.

Supplementary Fig. 1 shows the contribution of different diagnoses to AUD severity level 5. Seven out of ten people falling into this category were diagnosed with alcoholic liver cirrhosis only (K70; 40%), alcohol-induced psychosis, amnesia or another higher-order F10 diagnosis only (F10.5-9; 21%), or alcoholic neuropathy only (G62.1; 9.6%). The remaining sample had these or other diagnoses in combination.

Rather than using exclusive groupings, we decided to show the overlap of severity groupings for two reasons. With the overlaps, we were able to test our assumption that lower-order severities should be included in higher-order severities. Moreover, removing the people with higher-order severities from the lower-order severities would introduce a bias to the findings for lower-order severities. For example, if people with level 4/F10.3/4/alcohol withdrawal were excluded from level 3/F10.2/alcohol dependence, the latter group would likely appear healthier than they are. Information on exclusive groupings, however, is presented in Supplementary Table 1.

### Covariates

The following covariates were drawn from EHR data: (officially registered) sex (men, women), age (continuous) and employment status. While sex constitutes a time-invariant variable, the other two variables varied over the study period. Employment status was recorded by the SHI and categorized by us into four levels (employed, unemployed, retired, other – including student, migrant status). Within each year, the employment status with the most days (e.g., 300 days employed, 65 days unemployed ->employed) was kept. When different statuses had the same length, we applied a hierarchical selection (employed > unemployed > retired > other). This concerned *n* = 19 persons (0.08%) and *n* = 80 person-years (0.1%).

### Outcome: comorbidity

To validate AUD severity levels (aim 3), we examined same-year comorbidity for each person based on the Elixhauser comorbidity score (ECS). The ECS relies on diagnoses from inpatient and outpatient settings, with the same selection of the type of diagnoses applied as for AUD diagnoses (e.g., excluding ’symptomless’ diagnoses). The final ECS is a time-varying count variable that theoretically varies between 0 (no disease) and 31 (all diseases, including AUD).

### Statistical analyses

The total dataset contained up to 25 observations per person: up to five AUD severity levels per year over five years. For aims (1) and (3), we created two distinct subsamples; for aim (2), the total dataset was used.

For aim (1), we described people with different AUD severity levels using descriptive statistics such as averages (arithmetic means, medians) and measures of variation (interquartile range). For these analyses, we created subsample 1, which includes at most one observation per AUD severity level for each person – allowing up to five observations per individual. Since many patients experienced different AUD severity levels over the five-year period, subsample 1 captures the first year in which each severity level was recorded for each person. For example, if a person was diagnosed with AUD severity level 3 (F10.2, alcohol dependence) every year from 2017 to 2021, and additionally diagnosed with severity level 4 (F10.3/4, alcohol withdrawal) in 2019 and 2021, subsample 1 would include the 2017 record for level 3 and the 2019 record for level 4.

For aim (2), we calculated the annual administrative prevalence for each AUD severity level based on the total dataset. For this purpose, we divided the number of people in each level in each year by the number of people covered by the two SHI in that year.

For aim (3), we investigated whether AUD severity levels were associated with comorbidity. To this end, we created subsample 2, which includes at most one observation per person per year – resulting in up to five observations per individual across the study period. To capture intra-individual variation in AUD severity over time, subsample 2 retained only the highest AUD severity level recorded in each year for each person. For example, the person described above would contribute data for level 3 (F10.2, alcohol dependence) in 2017, 2018, and 2020, and for level 4 (F10.3/4, alcohol withdrawal) in 2019 and 2021.

We examined the association between AUD severity and ECS using generalized estimating equation (GEE) Poisson regression models for count data, specifying an exchangeable correlation structure to account for within-person correlation across repeated measures. These models estimate the population-averaged effect of AUD severity on ECS. All models controlled for year, sex, age group, and employment status in the respective year. For ease of interpretation, regression coefficients were exponentiated.

All analyses were performed with R version 4.4.2 [25].

## Results

### Description of AUD severity levels

Overall, *n* = 4,910,666 all-cause diagnoses (including *n* = 256,947 alcohol-specific diagnoses) from *n* = 21,954 persons between 2017 and 2021 were included in the analysis. The description was based on subsample 1, i.e., restricted to the first recorded AUD severity level for each person between 2017 and 2021.

Table 2 gives a descriptive summary of the study population by AUD severity level. The most frequent diagnosis was alcohol dependence (F10.2; severity level 3; *n* = 11,478), followed by harmful alcohol use (F10.1; severity level 2; *n* = 11,332), alcoholic liver cirrhosis (K70+; severity level 5; *n* = 6,003), acute alcohol intoxication (F10.0; severity level 1; *n* = 5,324) and alcohol withdrawal (F10.3/4; severity level 4; *n* = 3,016). This distribution was similar when examining the data for each year separately (results not shown).

**Table 2:** Description of study population by AUD severity

	0:any AUD	1:F10.0	2:F10.1	3:F10.2	4:F10.3/4	5:K70+
N	21,954	5,324	11,332	11,478	3,016	6,003
Sex (% female)	30.6%	29.4%	28.8%	29.2%	25.9%	32.1%
Age (Mean and IQR)	53.8 (42–66)	46.9 (32–59)	53.0 (41–64)	55.1 (45–65)	52.6 (43–61)	60.5 (51–72)
Education (%)						
employed	41.8%	38.2%	42.0%	36.3%	33.6%	34.9%
unemployed	25.1%	29.5%	26.8%	29.3%	33.7%	25.1%
retired	17.7%	12.3%	15.7%	18.5%	14.5%	26.1%
other	15.4%	20.0%	15.5%	15.9%	18.2%	14.0%
ECS (Mean and IQR)	4.2 (2–6)	3.5 (2–5)	4.3 (2–6)	4.5 (3–6)	4.9 (3–6)	5.7 (4–7)

Note. Study population covers all people insured with two statutory health insurances between 2017 and 2021 with at least one alcohol-specific diagnosis. Age, employment, and comorbidity was calculated for the first year of AUD severity grouping, if more than one year applied. ECS = Elixhauser comorbidity score (range: 0 [no comorbidity] to 31 diseases); IQR = interquartile range

In line with the hypothesized AUD severity levels, we found that overlaps with other diagnoses increased with rising AUD severity. Among people with F10.0 diagnoses (level 1), 59.7% also had at least one diagnosis from another (i.e., higher) AUD severity level. This percentage decreased to 55.2% for people with F10.1 diagnoses (level 2) but increased to 64.8% and 69.0% for people with F10.2 (level 3) and K70 + diagnoses (level 5), respectively. One exception was people with F10.3/4 (level 4) diagnoses, among whom 94.4% had at least one other alcohol-specific diagnosis.

**Fig. 1:**
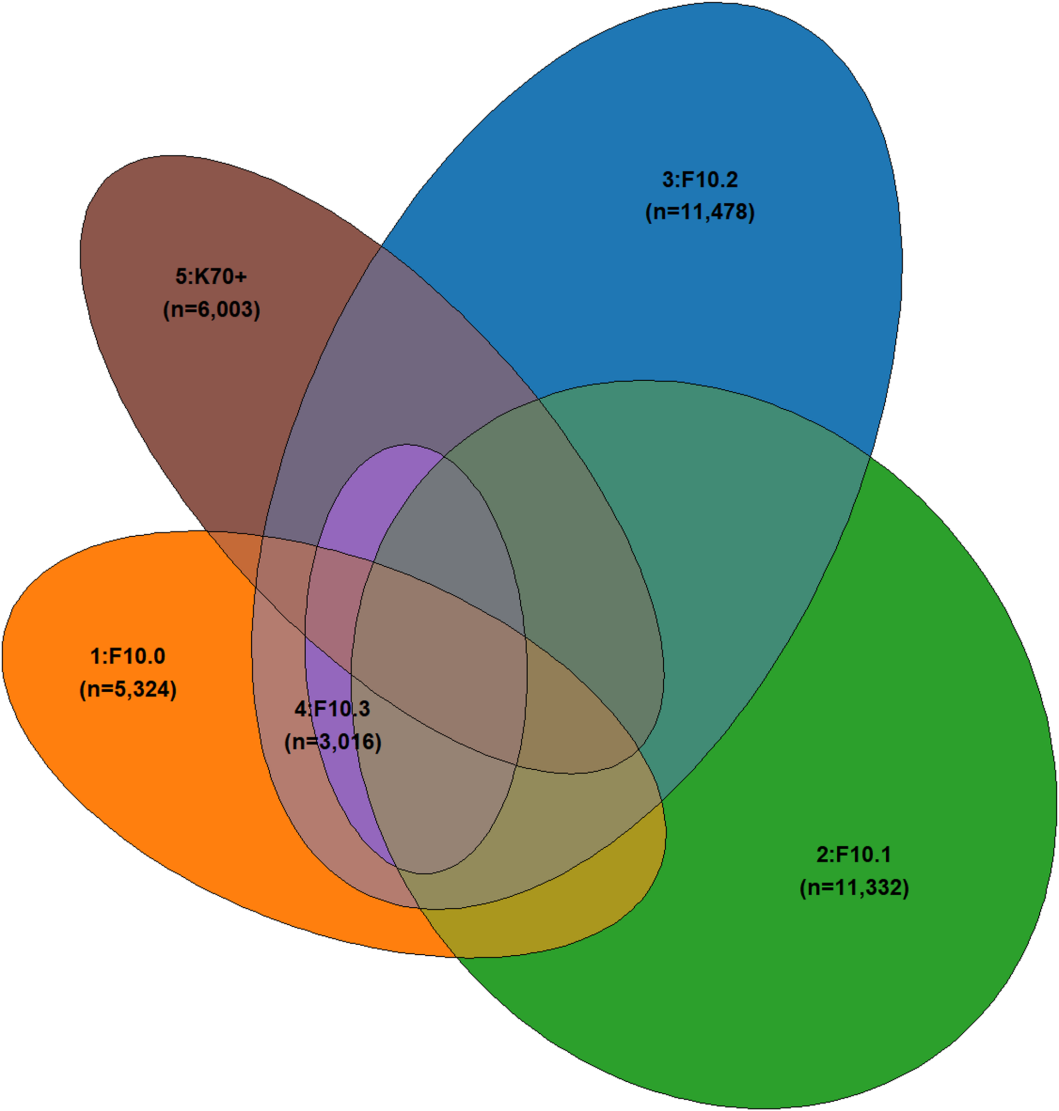
Venn diagram of *n* = 21,954 patients diagnosed in any setting/with any pattern. Five AUD severity levels are defined using alcohol-specific ICD-10 diagnoses, based on data between 2017 and 2021. Based on subsample 1 with data between 2017 and 2021

The overlap between F10.3/4 and other AUD severity groups is shown in the Venn diagram (Fig. 1), highlighting that F10.3/4 (level 4) is largely contained within F10.2 (level 3). Of the entire study sample (*n* = 21,954 with at least one alcohol-specific diagnosis), about one in four people (23.4%) were classified into both levels 2 (F10.1) and 3 (F10.2) at least once across the five years. Another major overlap was identified between levels 3 (F10.2) and 5 (K70+; 15.6%).

### Administrative prevalence of AUD severity levels

**Fig. 2:**
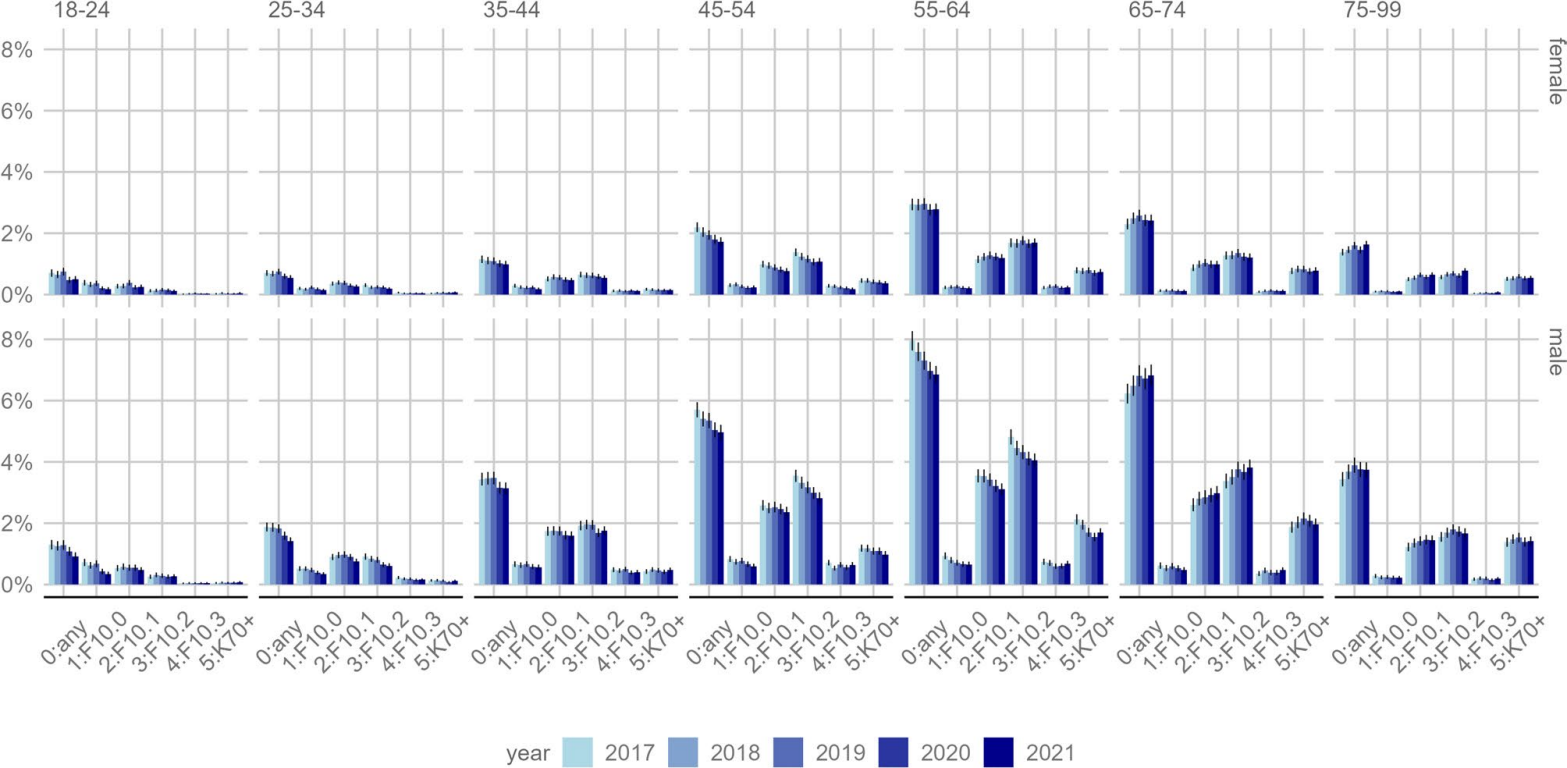
Administrative prevalence of AUD including 95% confidence intervals (error bars) by severity, sex, age group between 2017 and 2021

Across both sexes, the administrative prevalence of any AUD diagnosis (severity level 0) ranged between 2.9% (2020 and 2021) and 2.7% (2017 to 2019). Across the years, the average prevalence for AUD severity levels 1–5 was 0.4%, 1.3%, 1.5%, 0.2% and 0.7%, respectively. Only in the age group 18–24 years, AUD severity level 1 (F10.0) was more prevalent (0.43%) than other levels (2: 0.42%; 3: 0.21%; 4: 0.04%, 0.05%). As shown in Fig. 2, above-average prevalence figures for any AUD diagnosis were observed among men (4.2%; women: 1.6%) and among middle-aged and older adults (45–54: 3.6%; 55–64: 5.1%; 65–74: 4.3%) as compared to younger cohorts (18–24: 0.9%; 15–34: 1.2%; 35–44: 2.2%).

### AUD severity and comorbidity

A dose-response relationship between AUD severity and ECS is described in Table 2. The boxplots in Supplementary Fig. 2 illustrate that the median and interquartile range of the ECS increases with each incremental AUD severity level. Adjusting for sex, age group and year, AUD severity levels 2 to 5 are associated with 24% (95% CI: 21–27%), 32% (29–35%), 47% (43–51%) and 65% (61–69%) higher ECS as compared to AUD severity level 1. The full model results reported in Supplementary Table 2 show that there was a high within-person correlation of ECS (ρ = 0.708). The estimated scale parameter close to 1 (ϕ = 1.094) indicates low overdispersion. An alternative GEE model using an unstructured correlation structure yielded similar results (not shown).

**Fig. 3:**
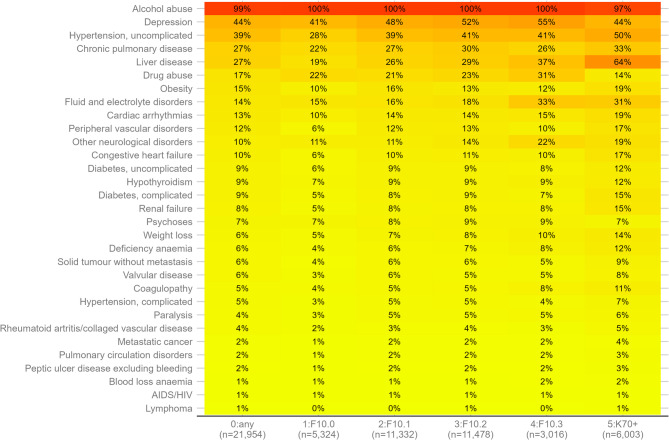
Percentage of study population meeting each Elixhauser comorbidity disease in the same year of AUD severity level classification. Based on subsample 2 with data between 2017 and 2021

As shown in Fig. 3, the most common comorbidities among people with any AUD were depression (44%), uncomplicated hypertension (39%), chronic pulmonary disease (27%) and liver disease (27%). With increasing AUD severity, the share of people with cardiovascular, pulmonary or liver diseases increased steadily. Conversely, the share of people without any diagnoses in the 30 disease groupings not specific to alcohol declined with greater AUD severity: for those with severity level 1 (F10.0; acute intoxication), 21.5% had no comorbidity according to the ECS definition and this percentage decreased to 9.2%, 6.6%, 4.6%, and 2.7%, for severity levels 2 to 5, respectively.

## Discussion

### Summary

This contribution proposes a novel description of AUD severity levels based on the ICD-10 diagnostic system. Relying on EHR data, we demonstrated that AUD can be approached using a dimensional definition by using a variety of ICD-10 diagnoses. Overall, we estimated that between 2.7 and 2.9% of the adult population with statutory health insurance in Hamburg, Germany, received an AUD diagnosis at least once per year. Among the five proposed AUD severity levels, the most prevalent were 2 (harmful use) and 3 (dependence), except in 18-24-year-olds (most prevalent: severity level 1 - intoxication). With increasing AUD severity, people experienced increasing comorbidity, in particular cardiovascular, pulmonary and liver diseases. Thus, our findings confirm the feasibility of our dimensional AUD severity approach and the hypothesized link with comorbidity.

### Limitations

First, we rely on billing data from SHI, which may be influenced by variations in the billing practices of physicians and treatment facilities. Possible biases may arise if diagnoses were selectively included in the billing data for reimbursement purposes (e.g., hospitals may receive more compensation for managing F10.1 as compared to F10.2). Although we are not aware of any specific reporting biases, the possibility of such bias cannot be entirely excluded. However, our administrative prevalence estimate for AUD is substantially lower than prevalence estimates from general population surveys (e.g., 9% among 18–64-year-olds: [26]), which is primarily driven by only a fraction of people with AUD seeking treatment [27]. This bias can also influence our severity grouping, as health problems are more likely to be diagnosed when people utilize health services.

Second, our definition is insensitive to identifying people with low AUD severity, even if they seek medical treatment for problems linked to alcohol use. The lowest threshold in our proposed AUD severity classification is an episode of acute intoxication. This threshold excluded a sizeable share of people with AUD, particularly young people, who drink heavily but have not (yet) developed any health problems requiring medical attention. As most people with heavy alcohol use are reportedly not routinely screened by general practitioners in Germany [28], including patients with elevated blood pressure due to heavy alcohol use [29], people with low AUD severity are expected to be underrepresented in our sample.

Third, there may be one source of distortion of the AUD severity distribution inherent to our approach: the practice of diagnosing alcohol withdrawal. While substantial withdrawal symptoms may also be experienced by people not entering detoxification treatment, it is unknown to what degree this population gets diagnosed with respective diagnoses (F10.3 or F10.4). Unlike alcoholic liver cirrhosis or other harms indicative of AUD level 5, diagnoses for AUD severity level 4 may be less important for clinical practice and may have been omitted. This may explain why fewer people were considered meeting the AUD severity level 4 compared to level 5.

### Implications

First, we have demonstrated the feasibility of our approach in a German sample of people with SHI. We confirmed our hypothesis of increased comorbidity with rising AUD severity, corroborating findings from a cross-sectional US survey [23].

To strengthen the confidence in our approach, we call for replications and external validation using data from other jurisdictions. We are aware of large cross-cultural differences in reporting AUD symptoms within Europe, resulting in large variations in AUD prevalence estimates [30]. Similarly, coding practices can vary largely across countries, driven by clinical guidelines or the degree of stigmatization. With AUD being one of the most stigmatized mental health conditions in high-income countries [31], it is possible that AUD diagnoses, in particular those indicating low AUD severity, may not be routinely registered in clinical practice. The robustness of our proposed dimensional approach against these biases could be demonstrated by replicating it across EHR data sources in different jurisdictions and with the ICD-11 diagnostic system.

Second, we want to stress that the distribution of AUD severity captured in EHR data differs substantially from the distribution in the underlying population. In the population, we expect the prevalence to decline with rising AUD severity, however, this is not observed in our data. The distribution of AUD severity in EHR data is expected to be determined by the need for treatment among those with AUD, the availability of health care services, and the willingness as well as capacities of medical professionals to screen for alcohol use and manage AUD. For example, if people with greater AUD severity were more likely to seek treatment [23, 32], they would be overrepresented in EHR data. Moreover, it was found that general practitioners in Germany and other European countries mainly recognize older patients with alcohol dependence and overlook about 2 in 5 patients with alcohol dependence [33].

Despite the potentially skewed distribution of AUD severity in our sample, we find a dose-response relationship between AUD severity and comorbidity, which supports the proposed dimensional approach. Thus, the potentially skewed distribution does not undermine the utility of this approach for identifying and comparing people with different AUD severity levels. Importantly, the identified temporal stability of different AUD severity levels is consistent with other publications on trends in alcohol use and harm in Germany [7, 34], thus supporting our approach.

Third, our proposed approach may allow further studies to conduct a nuanced analysis of AUD severity using EHR. For example, AUD severity has been linked to relapses [35], comorbidity and treatment in studies based on interview or survey data [23, 32]. However, studies relying on EHR data alone usually do not consider AUD severity, e.g., when predicting hospital re-admission risk (e.g. [9]), or for characterizing the role of physical comorbidity in in-hospital mortality [8]. By considering AUD severity in future studies, the different needs of people with AUD can be better approximated. This also entails accounting for the unstable nature of AUD [36] characterized by frequent remissions and relapses [35].

## Conclusions

AUD as a dimensional phenomenon can be addressed by using different types of ICD-10 diagnoses included in EHR data. In a sample of approximately 22,000 patients in Hamburg, Germany, we found that the proposed definition of AUD severity is closely linked to somatic comorbidities.

## Supplementary Information

Below is the link to the electronic supplementary material.

Supplementary Material 1

## Data Availability

The underlying data cannot be shared but the R codes are publicly available (https://github.com/jakobmanthey/PRAGMA_adminprev/).
