# Supplementary material for: Identifying levels of alcohol use disorder severity in electronic health records
Source: Subst Abuse Treat Prev Policy. 2025 Sep 8;20:36. doi: 10.1186/s13011-025-00670-w (PMC12418604; doi:10.1186/s13011-025-00670-w)
Supplement: Supplementary file 1 — Supplementary Material 1 [file 13011_2025_670_MOESM1_ESM.docx]

**SUPPLEMENTARY MATERIAL**

**Title:** Identifying Levels of Alcohol Use Disorder Severity in Electronic Health Records

**Authors:** Jakob Manthey, Carolin Kilian, Ludwig Kraus, Ingo Schäfer, Anna Schranz, Bernd Schulte

**Supplementary Figure 1:** Frequency of alcohol-specific diagnoses linked to AUD severity level 5. Illustrated are combinations of diagnoses that were registered among people with AUD severity level 5 at any time between 2017 and 2021 (n=6,003). Each area shows the relative size of all people with the respective diagnosis or combination of diagnoses. For example, the largest area indicates that 40% of the sample was diagnosed at least once with alcoholic liver cirrhosis but did not receive any other level 5 diagnosis (e.g., F10.5 or I42.6) during the observation period.


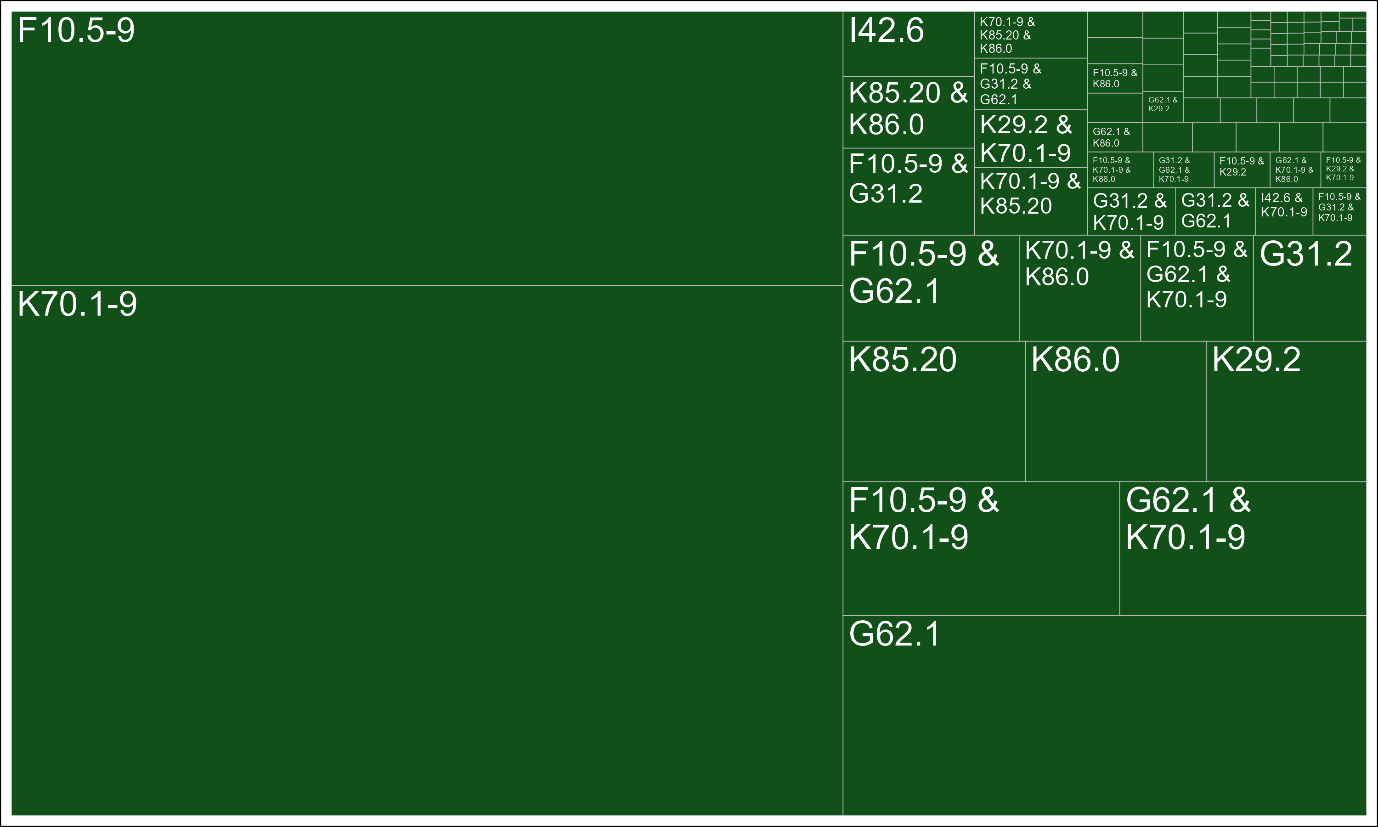


**Supplementary Figure 2:** Boxplots and jitter showing the distribution of the Elixhauser comorbidity score (ECS) by AUD severity level, based on subsample 2 with data between 2017 and 2021. Each dot represents one observation.


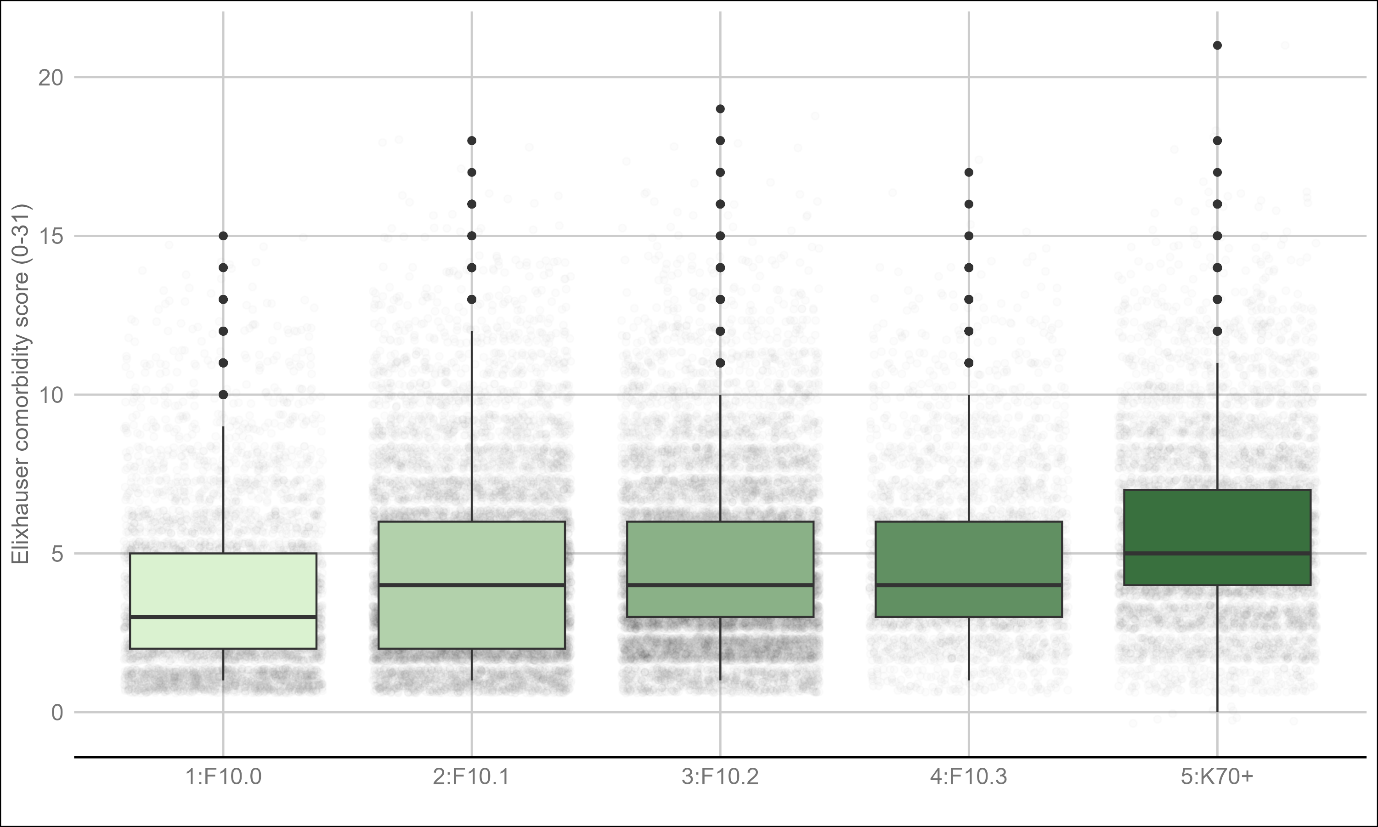


**Supplementary Table 1:** Description of study population by AUD severity (highest level only)

|  | **0:any AUD** | **1:F10.0** | **2:F10.1** | **3:F10.2** | **4:F10.3/4** | **5:K70+** |
| --- | --- | --- | --- | --- | --- | --- |
| N | 21,954 | 2146 | 5468 | 6689 | 1648 | 6003 |
| Sex  (% female) | 30.6% | 33.3% | 28.7% | 30.5% | 27.5% | 32.1% |
| Age  (Mean and IQR) | 53.8  (42-66) | 42.6  (25-58) | 51.4  (38-64) | 54.7  (44-65) | 51.2  (40-60) | 60.5  (51-72) |
| Education (%) |  |  |  |  |  |  |
| employed | 41.8% | 48.1% | 49.2% | 39.7% | 38.0% | 34.9% |
| unemployed | 25.1% | 20.7% | 22.7% | 27.0% | 31.2% | 25.1% |
| retired | 17.7% | 11.6% | 13.7% | 17.8% | 12.8% | 26.1% |
| other | 15.4% | 19.6% | 14.3% | 15.5% | 18.0% | 14.0% |
| ECS  (Mean and IQR) | 4.2  (2-6) | 2.6  (1-3) | 3.7  (2-5) | 4.2  (2-5) | 4.3  (3-5) | 5.7  (4-7) |
| Note. Study population covers all people insured with two statutory health insurances between 2017 and 2021 with at least one alcohol-specific diagnosis. Each person was assigned to the highest recorded AUD grouping. Age, employment, and comorbidity was calculated for the year of AUD severity grouping. ECS = Elixhauser comorbidity score (range: 0 [no comorbidity] to 31 diseases); IQR = interquartile range | | | | | | |

**Supplementary Table 2:** Results of general estimation equation Poisson regression models (exchangeable correlation structure) to link AUD severity with ECS (dependent variable), based on subsample 2 with data between 2017 and 2021.

| **Predictors** | **Incidence Rate Ratios** | **95% Confidence interval** | **p** |
| --- | --- | --- | --- |
| intercept | 1.92 | 1.85 – 2.00 | **<0.001** |
| sex (ref: female) | 0.94 | 0.93 – 0.95 | **<0.001** |
| age group (ref: 18-24) | | | |
| 25-34 | 1.08 | 1.03 – 1.12 | **<0.001** |
| 35-44 | 1.24 | 1.19 – 1.29 | **<0.001** |
| 45-54 | 1.46 | 1.41 – 1.52 | **<0.001** |
| 55-64 | 1.67 | 1.61 – 1.73 | **<0.001** |
| 65-74 | 1.93 | 1.86 – 2.01 | **<0.001** |
| 75-99 | 2.19 | 2.11 – 2.28 | **<0.001** |
| employment (ref: employed) |  |  |  |
| unemployed | 1.08 | 1.06 – 1.09 | **<0.001** |
| retired | 1.14 | 1.12 – 1.15 | **<0.001** |
| other | 1.04 | 1.03 – 1.06 | **<0.001** |
| year (ref: 2017) |  |  |  |
| 2018 | 1.02 | 1.01 – 1.03 | **<0.001** |
| 2019 | 1.03 | 1.03 – 1.04 | **<0.001** |
| 2020 | 1.04 | 1.03 – 1.05 | **<0.001** |
| 2021 | 1.05 | 1.04 – 1.06 | **<0.001** |
| AUD severity (ref: level 1) |  |  |  |
| level 2 | 1.24 | 1.21 – 1.27 | **<0.001** |
| level 3 | 1.32 | 1.29 – 1.35 | **<0.001** |
| level 4 | 1.47 | 1.43 – 1.51 | **<0.001** |
| level 5 | 1.65 | 1.61 – 1.69 | **<0.001** |
| Working correlation coefficient (ρ) | 0.708 |  |  |
| Scale parameter (ϕ) | 1.094 |  |  |
| N _patients_ | 21,954 |  |  |
| Observations | 56,899 |  |  |
